# Data analysis on near infrared spectroscopy as a part of technology adoption for cocoa farmer in Aceh Province, Indonesia

**DOI:** 10.1016/j.dib.2020.105251

**Published:** 2020-02-06

**Authors:** Agussabti, Rahmaddiansyah, Purwana Satriyo, Agus Arip Munawar

**Affiliations:** a Department of Agribusiness, Faculty of Agriculture, Syiah Kuala University, Banda Aceh, Indonesia; b Department of Agricultural Engineering, Faculty of Agriculture, Syiah Kuala University, Banda Aceh, Indonesia; c Agricultural Mechanization Research Centre, Syiah Kuala University, Banda Aceh, Indonesia

**Keywords:** Cocoa, Post-harvest, Technology, NIRS, Spectroscopy

## Abstract

Presented manuscript described data analysis on near infrared spectroscopy used as adopted and portable technology for cocoa farmers in Aceh Province, Indonesia. The near infrared spectroscopy (NIRS) assisted farmers in post-harvest handling especially for cocoa quality evaluation. This technology was used to determine moisture content (MC) and fat content (FC) of intact cocoa bean samples rapidly and simultaneously. Near infrared spectra data were acquired as absorbance spectrum in wavelength range from 1000 to 2500 nm with co-added of 32 scans for a total of 72 intact bulk cocoa bean samples. Spectra data can be used to predict MC and FC of intact cocoa beans by establishing prediction models and validate with actual MC and FC measured by means of standard laboratory procedures. Prediction performances were evaluated using several statistical indicators: coefficient correlation (r), coefficient of determination (R^2^), root mean square error (RMSE) and residual predictive deviation (RPD) index. Near infrared spectra data can be enhanced using spectra pre-treatment methods to improve prediction performances. Moreover, prediction models can be developed using principal component regression (PCR), partial least squares regression (PLSR) and other regression approaches. Ideal prediction models should have r and R^2^ above 0.75, RPD index above 2.0 and RMSE lower than its standard deviation (SD). Dataset were available as raw MS Excel format and *The Unscrambler* files as *.unsb extension.

**Table:** Specifications Table

Subject	Agricultural and Biological Sciences Post-harvest Technology
Specific subject area	Spectroscopy, technology adoption for farmer, non-destructive technology for cocoa quality evaluation
Type of data	Table Graph Spectroscopic data
How data were acquired	Near infrared spectral data of intact cocoa bean samples were collected and acquired using a self-developed portable near infrared spectroscopy (FTIR PSD i15). A total of 72 bulk intact cocoa bean amounted 50g per bulk were obtained from cocoa farmers in *Pidie Jaya*, Aceh Province, Indonesia. Cocoa beans were fermented for 3, 5 and 7 days in order to obtain different moisture content (MC) and fat content (FC) respectively. Spectra data were recorded as absorbance or Log (1/R) spectrum in wavelength range from 1000 to 2500 nm with co-added of 32 scans with resolution windows of 0.2 nm. Predicted MC and FC of intact cocoa bean samples were obtained simultaneously by performing calibration models using principal component regression (PCR) and partial least squares regression (PLSR) followed by cross validation respectively. On the other hand, actual reference data of MC and FC were measured by means of standard laboratory procedures as proposed by Refs. [1,2]. *Thermogravimetry* and *Soxhlet* methods were employed to measure actual MC and FC of cocoa samples respectively. Spectra data and actual MC and FC were then combined as one table in MS Excel that can be used for further analysis.
Data format	Raw Analysed Presented as *.xls* and *.unsb* extension file formats
Parameters for data collection	In cocoa trade markets, two main quality parameters considered are moisture content (MC) and fat content (FC). Both quality parameters were used as parameters for data collection and were predicted simultaneously using adopted NIRS technology.
Description of data collection	Spectra data were firstly subjected onto principal component analysis (PCA) and *Hotelling T*^*2*^ ellipse to detect potential outliers. Spectra data were then regressed with actual MC and FC to generate prediction models used to predict those both cocoa quality parameters. Predicted MC and FC were then compared with actual measured data in cross validation.
Data source location	Spectra data, actual moisture and fat content of intact cocoa beans were collected at the Department of Agricultural Engineering, Faculty of Agriculture Syiah Kuala University, Banda Aceh – Indonesia.
Data accessibility	Combined dataset are presented as MS Excel (.xlsx) and Unscrambler (.unsb) extension formats and available on this article. Dataset also can be found in Mendeley repository data: https://data.mendeley.com/datasets/7734j4fd98/1 or https://doi.org/10.17632/7734j4fd98.1

**Table:** 

**Value of the Data** • Spectra data of intact cocoa bean samples can be used to predict several quality parameters of cocoa beans simultaneously and rapidly bypassing standard laboratory procedures. • Provided dataset can be reanalysed and remodelled using different regression or spectra correction approaches. • Obtained models were benefited for cocoa manufacturers and industries for fast quality inspection of their cocoa products. • Spectral dataset can be corrected, enhanced using different pre-processing methods and applied onto prediction models. • Data generated from adopted NIRS technology proven to be useful for cocoa farmers in evaluating quality parameters.

## 1 Data

**Fig. 1:**
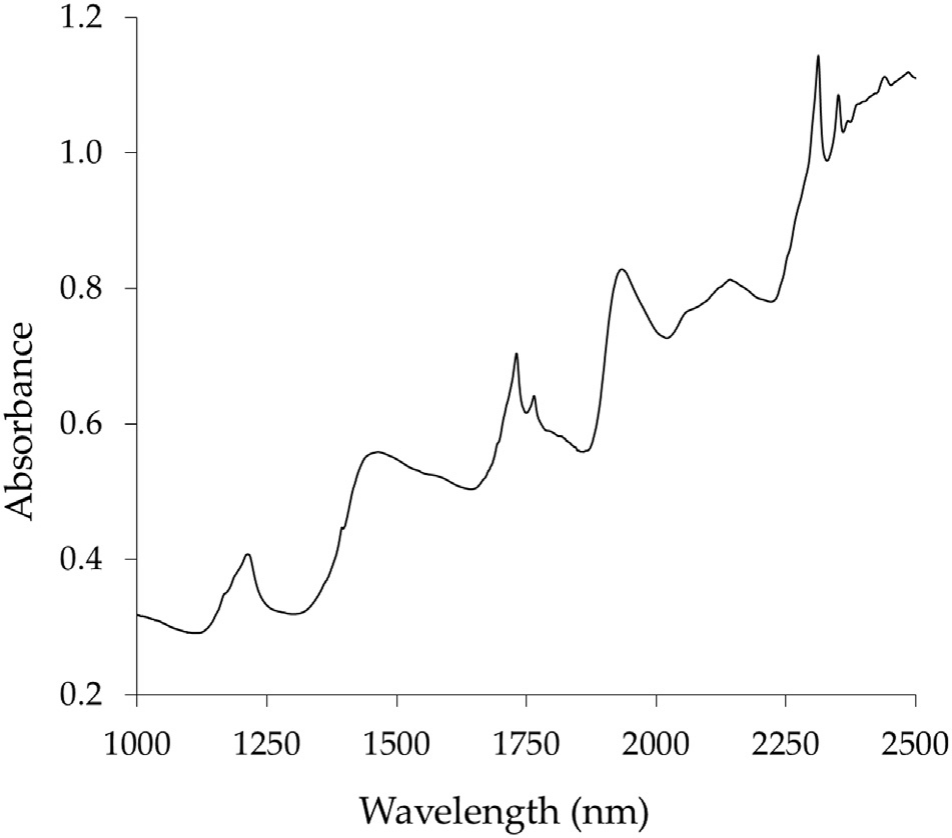
Typical near infrared absorbance spectrum of intact cocoa bean sample.

Typical spectra data feature of intact cocoa bean sample in near infrared region is shown in Fig. 1. Spectra data can be represented as reflectance, absorbance or transmittance spectrum in a function of energy (cm^−1^) or wavelength (nm) of the electromagnetic radiation. Spectra data of intact cocoa bean contains important information such as moisture content, fat content, carbohydrates, fibre and other quality parameters that can be revealed through calibration using several regression approaches. There are adequate regression methods available like multiple linear regression (MLR), support vector machine regression (SVMR), artificial neural network (ANN) based regression and two most commonly used as calibration: principal component regression (PCR) and partial least squares regression (PLSR).

It is recommended to pre-process spectral data prior to calibration and prediction models development in order to achieve better prediction performance [3,4]. Several pre-processing techniques are widely available such as spectra normalization, de-trending (DT), standard normal variate (SNV), spectra derivatives, multiplicative scatter correction (MSC), orthogonal signal correction (OSC) and others. Sometimes, those methods are combined in order to increase and improve spectra data [5].

Chemical information buried in cocoa bean samples corresponds to inner quality parameters like fat, fibre, phenol and carbohydrates. They consisted from structured molecular bonds of C–H–O, C–H, O–H, N–H and R–O–H for alcohol. Specific wavelengths in near infrared region are corresponded to certain chemical properties and quality parameters of cocoa samples from which represents the amount of light reflected, absorbed or transmitted [6].

**Fig. 2:**
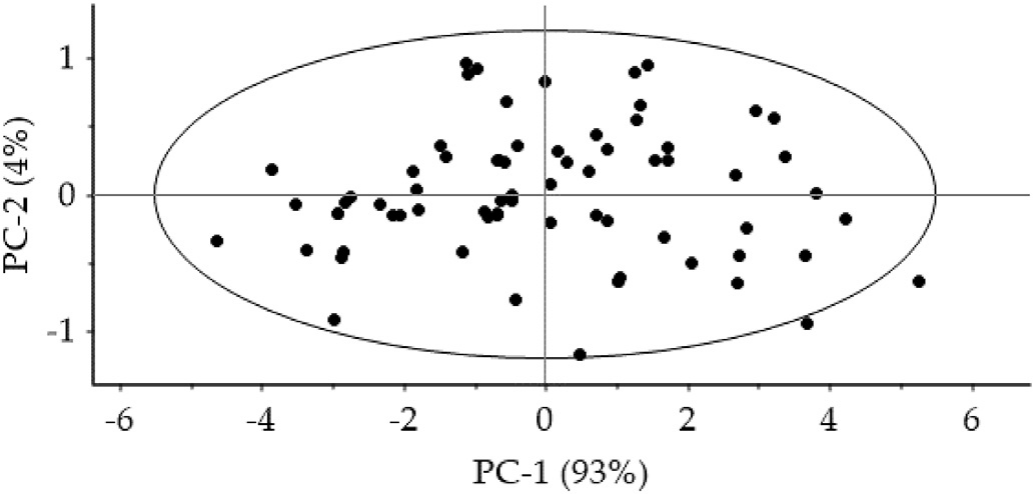
Spectra data projected onto principal component analysis (PCA) and *Hotelling* T^2^ ellipse.

In NIRS applications and practices, there are some problems related to the selection of representative sample datasets used for calibration, cross validation and independent validation. Splitting a large dataset into calibration and validation subsets normally varied out by splitting onto 70:30 for those subsets respectively. NIRS users also found difficulties in identifying and detecting samples that are somehow considerably different from the majority of the remaining samples from which known as outliers. Based on literatures and common NIRS practices, outlier data can be identified by projecting spectra data onto principal component analysis (PCA) and applying *Hotelling* T^2^ ellipse as presented in Fig. 2. Data lies outside the ellipse needs to be checked due to their potential outliers.

**Fig. 3:**
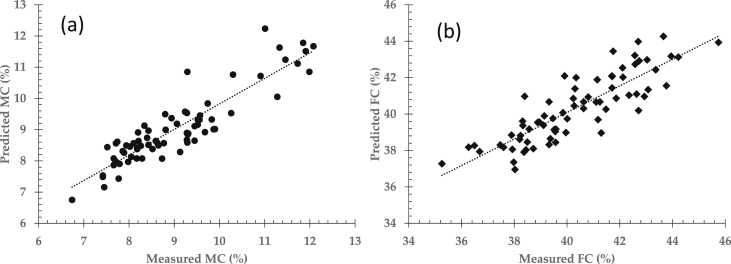
Prediction performance of moisture content (a) and fat content (b) by means of principal component regression (PCR) approach.

**Fig. 4:**
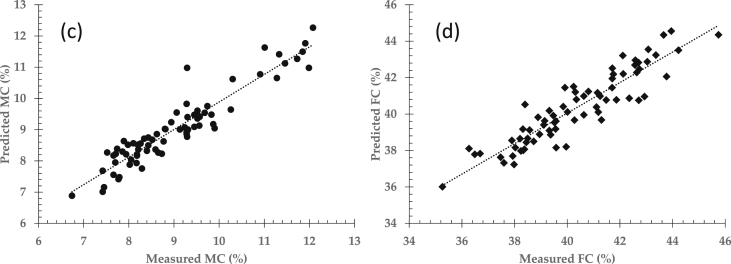
Prediction performance of moisture content (c) and fat content (d) by means of partial least squares regression (PLSR) approach.

To determine inner quality parameters in intact cocoa bean samples, NIR spectra data were regressed with actual measure moisture and fat contents using mentioned regression approaches. The two most common approaches are principal component regression (PCR) and partial least squares regression (PLSR) since these both regression methods were normally used in the emerging field of NIRS till date [7,8]. Both PCR and PLSR methods seek to find best correlation between near infrared spectra data and inner quality parameters i.e moisture content (MC) and fat content (FC) of cocoa bean samples as presented in Table 1, Table 2. To evaluate prediction performance, predicted results of moisture and fat content were then compared to the actual measured MC and FC as shown in Fig. 3 and Fig. 4 using principal component regression and partial least squares regression approaches respectively.

**Table 1:** Prediction performance between principal component regression (PCR) and partial least squares regression (PLSR) for moisture content prediction.

Regression approach	Statistical indicators				
	Factor	R^2^	r	RMSE	RPD
PCR	7	0.82	0.90	0.54	2.37
PLSR	7	0.88	0.94	0.42	3.05

R^2^: coefficient of determination, r: correlation coefficient, RMSE: the root mean square error, RPD: residual predictive deviation.

**Table 2:** Prediction performance between principal component regression (PCR) and partial least squares regression (PLSR) for fat content prediction.

Regression approach	Statistical indicators				
	Factor	R^2^	r	RMSE	RPD
PCR	7	0.73	0.85	1.11	1.94
PLSR	7	0.84	0.91	0.82	2.62

R^2^: coefficient of determination, r: correlation coefficient, RMSE: the root mean square error, RPD: residual predictive deviation.

Prediction performance can be enhanced and improved by correcting spectral data prior to calibration development. It is strongly recommended to pre-process spectral data in order to generated and achieve more accurate and robust prediction results. Table 3 showed a comparison among different spectra correction methods in determining moisture and fat content in intact cocoa bean samples. Prediction models were established using partial least squares regression followed by cross validation method.

**Table 3:** Comparison among different spectra correction methods to the prediction performance of inner quality parameters in cocoa bean samples using partial least squares regression approach.

Quality parameters	Correction method	Statistical indicators				
		Factor	R^2^	r	RMSE	RPD
Moisture content	MN	5	0.90	0.95	0.40	3.18
	Smoothing	5	0.91	0.95	0.39	3.27
	SNV	5	0.91	0.95	0.38	3.37
	EMSC	5	0.92	0.95	0.37	3.46
Fat content	MN	5	0.95	0.98	0.45	4.78
	Smoothing	5	0.90	0.95	0.68	3.16
	SNV	5	0.97	0.98	0.38	5.66
	EMSC	5	0.98	0.99	0.27	7.96

EMSC: extended multiplicative scatter correction, MN: mean normalization, R^2^: coefficient of determination, r: correlation coefficient, RMSE: the root mean square error, RPD: residual predictive deviation. SNV: standard normal variate.

**Fig. 5:**
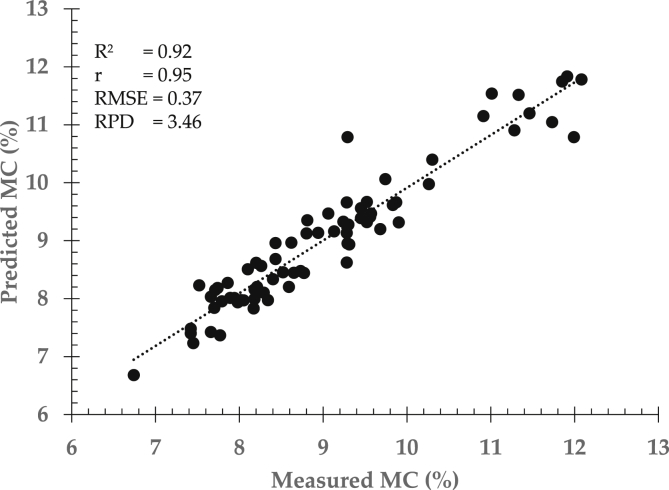
Prediction performance for moisture content (MC) determination using enhanced EMSC spectra data and partial least square approach.

**Fig. 6:**
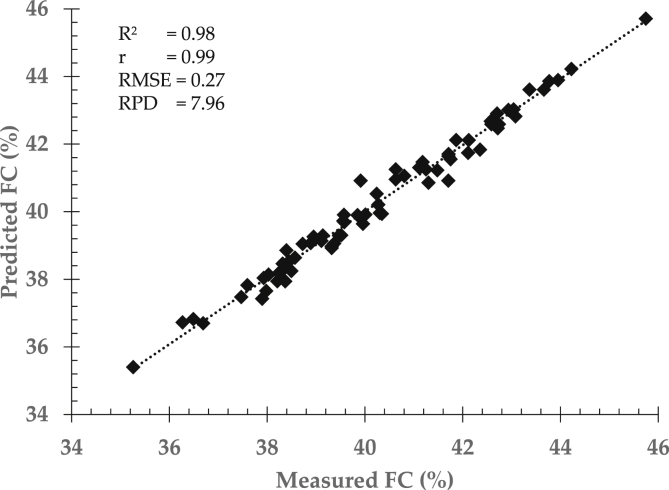
Prediction performance for fat content (FC) determination using enhanced EMSC spectra data and partial least square approach.

Prediction accuracy and robustness normally improved when the models are constructed using enhanced spectral data. Yet, prediction performance may vary depends on spectra pre-processing method as shown in Fig. 5 for moisture content and fat content respectively. Spectra corrections can also be combined to increase prediction accuracy and robustness. However, combination selection must be chosen carefully to avoid overfitting and removal important information of quality attributes (see Fig. 6).

## 2 Experimental design, materials, and methods

### 2.1 Spectra data acquisition

Near infrared spectral data of intact cocoa bean samples were acquired using a portable near infrared spectroscopy (FTIR PSD i15) in wavelength range from 1000 to 2500 nm. A total of 72 bulk intact cocoa bean amounted 50g per bulk were obtained from cocoa farmers in *Pidie Jaya*, Aceh Province, Indonesia. Cocoa beans were fermented for 3, 5 and 7 days in order to obtain different moisture content (MC) and fat content (FC) respectively. Spectra data were recorded as absorbance or Log (1/R) with co-added of 32 scans and resolution windows of 0.2 nm [9]. Spectra data were recorded and saved in two different file formats as *.csv and *.spa. For each spectra measurement, sample labelling was required automatically before acquisition to differ cocoa bean samples. Furthermore, predicted MC and FC of intact cocoa bean samples were obtained simultaneously by performing calibration models using principal component regression (PCR) and partial least squares regression (PLSR) followed by cross validation respectively. Data used for testing and validating models were obtained from calibration datasets from which cross validation method is employed [10].

### 2.2 Moisture and fat content measurements

After spectral data acquisition were completed, all cocoa bean samples were taken directly for actual inner quality parameters (moisture and fat content) measurement. Actual referenced moisture content was determined using a *gravimetry* method and measured in duplicate, then averaged. The moisture content of cocoa bean samples is expressed in % dry bulb. On the other hand, actual fat content was determined using *Soxhlet* method. Ten gram of cocoa sample was mixed in the tube with maximum of 150 ml n-hexane and extracted in *Soxhlet* apparatus at temperature of 95 °C for 6 h [11]. Fat content was then determined by evaporating those solvent using rotary evaporator until fat liquid is remained in the tube. Then, it dried at temperature of 105 °C for about 30 minutes. Fat content was expressed in % fat content and measured also in duplicate then averaged. Descriptive statistics of actual measured moisture and fat content of cocoa bean samples are shown in Table 4.

**Table 4:** Descriptive statistics of actual measured quality parameters of cocoa bean samples.

	Moisture Content (%)	Fat Content (%)
# of Sample	72	72
Mean	9.04	40.32
Max	12.08	45.75
Min	6.74	35.26
Range	5.34	10.49
Std. Deviation	1.28	2.15
Variance	1.63	4.64
RMS	9.12	40.38
Skewness	0.84	0.10
Kurtosis	0.07	−0.46
Median	8.79	40.13
Q1	8.09	38.55
Q3	9.56	41.93

### 2.3 Sample outlier detection

In many spectroscopy practices, there are some spectra data of related samples somehow considerably different from the majority of the remaining samples from which known as outliers. They can be found in the sample datasets used for model construction and validation, or arise among new samples during the use of those models for independent prediction.

Several methods were widely employed to detect and identify outliers, among of them is the combination of principal component analysis (PCA) and Hoteling T^2^ ellipse. It used to define statistical boundaries assuming a normal distribution of scores of the PCA. First, raw spectral data of cocoa samples obtained from the NIRS instrument are projected onto PCA map. Then, Hoteling T^2^ ellipse is applied to this PCA projection. Typically, outliers are identified as samples found outside the ellipse confidence limit [12,13]. The ellipse usually established at the level of 95%. Another method that also can be used for outlier detection is the *Mahalanobis* distance and the spectral residual applied to raw spectra data. Raw spectra data refers to the original data before spectra correction and enhancement.

### 2.4 Spectra data corrections

To achieve robust and accurate prediction results, spectra data need to be corrected and pre-processed prior to prediction model development. To compensate some noises on spectra data, smoothing with *Savitsky-Golay* algorithm can be applied to eliminate noises. Then, mean normalization (MN) can be also a choice as spectra correction method to remove background irrelevant information and normalize spectral data. Moreover, standard normal variate (SNV) is a good choice to pre-process and enhance spectra data as this method is widely employed beside multiplicative scatter correction (MSC) either as full MSC or as extended (EMSC). Both these methods proven to be effective and improve prediction performance.

### 2.5 Prediction models

The main part of NIRS applications is to establish models used to predict desired quality parameters simultaneously of cocoa samples through a process called as calibration. In many NIRS cases, most classical linear algorithms such as multiple linear regression (MLR), principal component regression (PCR) and partial least squares regression (PLSR) have proven to be effective. In comparison, PLSR normally generated a better prediction results compared to the other two. Partial least square method can simultaneously decompose the spectral data (X-variable) and the actual measured quality parameters data (Y-variable). It considers the relationship between the two in the decomposition, strengthen the corresponding calculation and ensuring the best correction model [14,15]. Therefore, the PLSR method continue to be the workhorse for regression in NIRS applications.

The original work of PLSR is a linear method which assuming a linear relationship of the modelled quality parameters or concentrations as a function of the near infrared spectral data variations. Weak nonlinearities may be solved by increasing the number of factor or also known as latent variables (LVs) included in the PLSR. Another most common method as NIRS user preference for constructing and developing prediction models is principal component regression (PCR). It is a similar multivariate regression method works based on principal component analysis and multiple linear regression.

In recent years, prediction models in NIRS can be constructed using a non-linear regression approach like support vector regression and artificial neural networks. It was carried out due to the fact that there is a certain nonlinearity between the spectral data and the quality parameters data. Thus, nonlinear correction model must be established for the unique nonlinear characteristics of the system.

The prediction performances were evaluated by means of these following statistical indicators: the coefficient of determination (R^2^) and correlation (r) between predicted and measured quality parameters (moisture and fat content), prediction error which is defined as the root mean square error (RMSE) and the residual predictive deviation (RPD) or some papers called it as the relative predictive determinant [8,16,17]. The RPD defined as the ratio between standard deviation (SD) of the actual measured value of moisture content (MC) and fat content (FC), and the RMSE of predicted quality parameters. Last but not least, optimum number of factor or known as latent variables (LVs) were chosen based on the minimization of RMSE during cross-validation.
